# School girls and childbearing motives: A randomized clinical trial through hidden curriculum

**DOI:** 10.18502/ijrm.v17i12.5805

**Published:** 2019-12-30

**Authors:** Zeinab Oshrieh, Afsaneh Keramat, Mohammad Shariati, Najmeh Tehranian, Elham Ebrahimi, Mohammad Effatpanah

**Affiliations:** ^1^ Student Research Committee, School of Nursing and Midwifery, Shahroud University of Medical Sciences, Shahroud, Iran.; ^2^ Reproductive Studies and Women’s Health Research Center, Shahroud University of Medical Sciences, Shahroud, Iran.; ^3^ Tehran University of Medical Sciences, Tehran, Iran.; ^4^ Department of Reproductive Health and Midwifery, Tarbiat Modares University, Tehran, Iran.; ^5^ Department of Reproductive Health, School of Nursing and Midwifery, Tehran University of Medical Sciences, Tehran, Iran.; ^6^ Department of Psychiatry, Ziaeian Hospital, School of Medicine, Tehran University of Medical Science, Tehran, Iran.

**Keywords:** Childbearing, Motive, School, Adolescents, Intention.

## Abstract

**Background:**

Childbearing motives are considered as the primary stimulus of fertility and the importance of making fertility decisions in humans.

**Objective:**

The aim of this study is to determine the effect of a new form of interactional program on the childbearing motives of students.

**Materials and Methods:**

According to a well-defined, single-blind randomized controlled trial, we selected eight government schools in Tehran. Students in the age range of 7-12 yr and 16-18 yr (130 students in each school with a total number of 260) were selected for a series of intervention from storytelling to free discussion on a special subject through the concept of hidden curriculum. We evaluate the outcome with pretest-posttest based on the Miller childbearing motives questionnaire. One month after the last intervention, final evaluation took place.

**Results:**

The findings showed that after an intervention conducted in the best way, all positive scores were promoted while the negative ones declined. One the other hand, no matter what really the participant's groups were, their total intention score got better. In this way, the total positive scores were significantly increased in the intervention groups (p = 0.000) Also, the students in high school significantly improved in positive scores and the negative score decreased in them.

**Conclusion:**

This study showed that the fundamental childbearing motives even with small interventions can be improved. Our intervention could improve the positive childbearing motives among school girls. In this regard, the role of some confounding factor such as the role of some confounding factors such as religious beliefs in family, maternal education is most important.

## 1. Introduction

Childbearing is the most important component of population growth (1). Childbearing motives are considered as the primary stimulus of fertility and the importance of making fertility decisions in humans (2). Fertility and childbirth are unique factors in population growth worldwide so that they can change the demographic structure in countries (3). The tendency and general desire of human beings to have children is one of the most important components of development in societies and is a pivot of sustainable development for countries with low population substitutions (4). In recent years, there has been a dramatic change in the population of the world, so that one of the most important changes in this regard is the declining infertility (5). Along with these changes, the fertility rate in Iran has also decreased. The results of censuses and statistics in Iran show that the total fertility rate declined from 7.7 children per woman in 1967 to 1.6 in 2011. These figures indicate that our country stands at the replacement level (5, 6). One of the target group that according to them have a significant effect on the demographic changes are school girls. According to the demographic statements, every country needs to collect specific data about the factors that may affect the intentions of adolescent girls (7, 8).

The teenage period is the stage from childhood to adulthood in which many patterns of life are learned and established (9). Because of limited access to reproductive health education and cares, adolescents have to enter in childbearing life with relatively low reliance on contraceptive selections, pregnancy intentions, and high-risk behavior protections (10). In the meantime, the role of education should not be overlooked. According to numerous studies, the learning power of people at lower ages is higher (11). Countries were encountered to decrease, turned into a variety of training approaches that can create positive attitudes in fundamental demographic groups such as school pupils. One of the most recent interventions is the training approach through the hidden curriculum (12-14). Usually, sociologists, educators, and psychologists often use the concept of the hidden curriculum to describe the informal school system. The hidden curriculum is not written anywhere nor does it teach any teacher. But the school's educational environment, with all its features, teaches it. Students are incidentally subject to something that has never been spoken about regardless of how well-educated, competent the school teachers are and how progressive the curriculum is. They are, by the way, influenced by the hidden school curriculum, a special approach to life, and a special attitude toward the subject. Its main function is social control. They believe that students are confronted with the hidden curriculum in their own way. In other words, the school, through the hidden curriculum, implicitly prepares students to accept their future social status (15, 16).

According to the best of knowledge, the data about the childbearing motivation in this age group is limited in our country. These studies were done without any need assessment with a traditional method and the aims of these studies were specially about the puberty health but not about the subject of childbearing motives (17-19). So that few interventions developed in this area. This article shows the study design and protocol of our intervention.

The aim of this study is to determine the effect of a new form of interactional program on the childbearing motives of students.

## 2. Materials and Methods 

**Figure 1:**
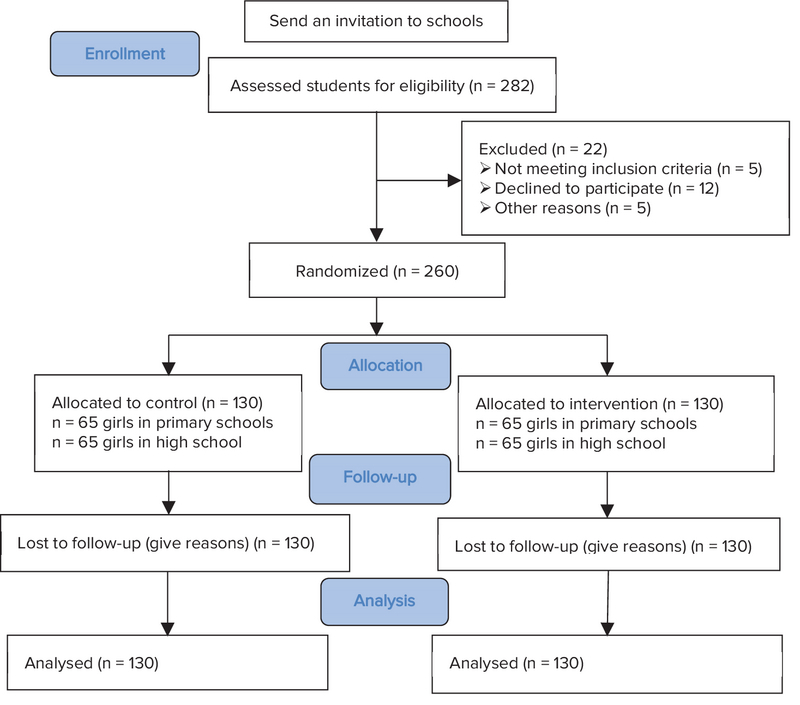
Consort diagram.

According to a well-defined, single-blind randomized controlled tria,l we evaluated the effects of our intervention. Figure 1 shows the steps of eligibility assessment and the study progress based on the Consort. We used a pretest-posttest intervention design in eight governmental schools in Tehran (four elementary and four high schools). The sample size was determined based on “n = (Z1 + Z2) 2 (2S2)/d2” formula. At this level, we recruited 260 students. At the level of school selection, Tehran city was divided into four sections (according to the geographical division), and in each part, one primary and one high schools were randomly selected. In the selected schools, the assignment of classes to the intervention and control groups was randomized with a drawing approach. We selected all students who were in the age range of 7-12 yr and 16-18 yr for elementary and high schools, respectively, and willing to participate in the study. If any of these students refused to follow the intervention or were absent for two sessions, they were excluded from the study.

The consolidated standards of reporting trials:

Z1 = Z value corresponding to type I (significance level) = 1.96

Z2 = Z value corresponding to type II errors = 0.84

S = Estimate of standard deviation of the sample

d = Distance from mean to one side of the range that was considered 0.4 s.

10% potential dropout at one month

### Intervention

In elementary schools, the intervention was conducted in two days. At the beginning of each session, the participants responded to the pretest questions. After collecting the questions, the type of intervention and expected tasks were described, and the intervention was implemented. Subsequently, posttest questions were provided to the participants. The first day of the intervention began in the form of a group discussion, in which an educational clip was displayed and after that student were asked to discuss it. On the second day of intervention (a week later), the method of storytelling-rethinking was used in which a story was told (its content would be presented in the supplementary data) and participants were asked to think about it and remove the main concepts of it. One month after the last intervention, a third evaluation took place. Miller's childbearing motive questionnaire was used for the evaluation.

Because of the higher level of cognition in high school girls, the content of the interventions in this group was slightly different from the other groups. The same as the previous one, the way of the evaluation was before and after questions. On the first day, an unfinished story was told. The students were divided into three groups and were asked to complete the story. On the second day (a week later), students told a memory in our specific subject and after that, they discussed it with each other. Like mentioned earlier, the third evaluation was done one month later.

### Questionnaire

The childbearing motivation questionnaire was developed by Warren Miller (1995) and used for assessing the strong and weak aspects of childbearing motivation among participants. It offers comprehensive data on individual's childbearing desire. This questionnaire consists of two dimensions. Positive motives that include the pleasure of pregnancy, birth, and childhood (6 questions), traditional view (6 questions), satisfaction from parenting (6 questions), need and survival (5 questions), and the instrumental use of the child (11 questions) The other dimension relates to negative motives which include fear of being pregnant (7 questions), stress of parents (8 questions), and childcare challenges (4 questions).In this study, we use the Persian version of this questionnaire (20). The validity of the questionnaires was confirmed by Cronbach's alpha coefficient of 98%. In the part of reliability assessment, the correlation coefficient of 91% was obtained. In this version, the original questionnaire was translated and also expanded. Items were added to both sections based on the qualitative research conducted in Iran. In total, there are 26 items to measure positive motives and 11 in the negative section. With multiple questions, we were able to explore the structure of childbearing motives. In the control group at each level, the participant only responded to questions in two different times.

### Ethical consideration

A written consent was received from all participants. Ethical code is IR.SHMU.REC.1395.96 obtained from the Shahroud University of Medical Sciences.

### Statistical analysis

The data analysis was conducted based on SPSS, version 18. While the analytical statistics (such as paired t-test and logistic regression) were used to get to the conclusion about the efficacy of intervention, and the descriptive statistics (i.e., mean, mean difference, frequency, and frequency percent) are used to give more information about some particular data.

## 3. Results

Table I shows variation in demographic characteristics and the possible factors affecting fertility motivation among participants in the study. These factors have been extracted based on a comprehensive overview of basic research in this area (21).

Table II shows the change in the score of the positive and negative childbearing motives in school girls. Based on the findings of this table, at the elementary level, the greatest change in positive motivation has occurred in “satisfaction with parenting.” In high school students, after the intervention, participants showed a significant increase in the score of all positive motive domains, except for the use of a “child's tool.” Although the scores of negative motive domains were decreased in both groups after the intervention, this decline was not statistically significant.

Table III formulates the school children's childbearing motivation based on some possible demographic and potential variables.

**Table 1:** Characteristics of study participants


**variable**	**Elementary school N = 130**		**High school N = 130**	
	**Control n (%)**	**Intervention (%)**	**Control n (%)**	**Intervention n (%)**
Mother's level of education				
Illiterate	0 (0)	1 (1.53)	2 (3.07)	0 (0)
Primary school	8 (12.3)	5 (7.7)	3 (4.61)	9 (13.85)
High school	19 (29.23)	23 (35.38)	16 (24.61)	27 (41.54)
University	38 (58.47)	36 (55.39)	44 (67.7)	29 (44.61)
Father's level of education				
Illiterate	1 (1.53)	0 (0)	0 (0)	2 (3.07)
Primary school	5 (7.7)	3 (4.61)	6 (9.23)	3 (4.61)
High school	17 (26.15)	11 (16.7)	7 (10.76)	5 (7.7)
University	42 (64.61)	51 (78.46)	52 (80)	55 (84.61)
Mother's job				
Housewife	37 (56.9)	26 (40)	24 (36.92)	23 (35.38)
Employee	28 (43.1)	39 (60)	41 (63.08)	42 (64.62)
Father's job				
Government Employee	35 (53.84)	44 (67.7)	34 (52.3)	23 (35.38)
Self-employed	30 (46.16)	21 (32.3)	31 (47.7)	42 (64.62)
Number of sisters or brothers				
1-2	45 (69.23)	38 (58.46)	48 (73.84)	34 (52.3)
3 ≤	20 (30.7)	27 (41.54)	17 (26.16)	31 (47.7)
Religious beliefs in family				
Yes	39 (60)	38 (58.46)	43 (66.15)	47 (72.31)
No	26 (40)	27 (41.53)	22 (33.85)	18 (27.69)

**Table 2:** Mean score and standard deviation of childbearing motive's score, before and after the intervention in the groups


**Childbearing motive**		**Control**			**Intervention**		
	**Level 1**	**Level 2**	**P-value**	**Level 1**	**Level 2**	**P-value**
Elementary school N = 130							
	Pleasure of childbearing	16.51 ± 3.1	17.12 ± 2.73	0.45	17.23 ± 3.5	17.43 ± 1.2	0.33
	Traditional view	23.12 ± 2.9	24.1 ± 1.67	0.01	26.23 ± 1.2	28.33 ± 2.3	0.000
	Satisfaction with parenting	17.71 ± 1.2	17.92 ± 2.7	0.28	19.23 ± 3.1	25.36 ± 1.9	0.000
	Feeling need for survival	12.98 ± 0.9	13.2 ± 1.53	0.16	15.21 ± 1.6	16.1 ± 3.4	0.03
<brow>-5</erow> Positive	Instrumental use of child	11.84 ± 3.8	12.26 ± 1.33	0.20	14.21 ± 2.6	15.6 ± 1.67	0.002
	Fear of being parent	19.35 ± 2.3	19.16 ± 1.7	0.70	17.13 ± 1.6	14.15 ± 1.36	0.1
	Parenting stress	19.79 ± 3.8	19.36 ± 3.6	0.74	21.43 ± 3.6	19.11 ± 1.15	0.1
<brow>-3</erow> Negative	Child's care challenges	18.66 ± 3.4	17.78 ± 2.5	0.95	16.58 ± 3.2	13.01 ± 1.93	0.1
High school N = 130							
	Pleasure of childbearing	19.53 ± 2.5	22.12 ± 1.87	0.000	18.87 ± 1.35	24.96 ± 2.68	> 0.001
	Traditional view	19.11 ± 3.1	19.73 ± 2.6	0.10	18.95 ± 2.4	23.1 ± 1.67	> 0.001
	Satisfaction with parenting	23.36 ± 0.12	27.14 ± 4.32	0.000	25.12 ± 1.76	29.1 ± 2.03	> 0.001
	Feeling need for survival	22.11 ± 1.5	27.16 ± 1.35	0.000	19.9 ± 3.2	26 ± 1.67	> 0.001
<brow>-5</erow> Positive	Instrumental use of child	15.39 ± 3.9	13.12 ± 2.76	0.99	17.12 ± 3.8	18.1 ± 1.67	0.97
	Fear of being parent	18.19 ± 2.39	17.13 ± 1.71	0.99	18.12 ± 1.78	16.33 ± 1.5	0.1
	Parenting stress	17.13 ± 3.8	13.55 ± 1.23	0.1	17.11 ± 2.1	13.21 ± 1.1	0.1
<brow>-3</erow> Negative	Child's care challenges	17.77 ± 2.17	16.47 ± 1.25	0.99	23.17 ± 3.9	17.8 ± 2.1	0.1
Data presented as Mean ± SD. The significance area was considered in the level of 0.05							
P-value was determined based on paired t-test

**Table 3:** Regression model based on the childbearing motive's score and the demographic variables


**Childbearing motive**	**Demographic variable**	**Coefficient**	**Confidence interval 95%**	**P-value**
	Mother's level of education	-0.780	(0.916-0.456)	0.001
	Father's level of education	-0.612	(0.857-0.370)	0.001
	Mother's job	-0.237	(-0.371-0.178)	0.001
	Father's job	-0.312	(-0.268-0.132)	0.01
	Number of sisters or brothers	0.217	(-0.336-0.129)	0.03
<brow>-6</erow> Positive	Religious believes in family	0.821	(0.936-0.632)	0.001
	Mother's level of education	0.761	(0.815-0.312)	0.001
	Father's level of education	0.501	(0.621-0.358)	0.01
	Mother's job	0.643	(0.795-0.573)	0.001
	Father's job	-0.527	(-0.651-0.257)	0.001
	Number of sisters or brothers	0.668	(0.803-0.513)	0.001
<brow>-7</erow> Negative	Religious beliefs in family	-0.740	(-0.781-0.612)	0.001
Logistic regression test			

## 4. Discussion

The finding of this study showed that an intervention conducted in the form of interaction between students can promote scores of childbearing motives, whereas the negative ones declined. One the other hand, no matter what really the participant's group is, their total intention score got better. The childbearing motive is the most important factor which can affect the fertility behavior (1). In each country, the population of girls and their fertility behaviors have a very significant impact on the future image of the population. In this way, as one of the incentive policies, the countries that have a potential negative growth in their population attempt to correct the beliefs of this group of people (2). This study was designed to investigate the effectiveness of a learning intervention through the hidden curriculum that administered in the schools on the positive and negative motives of school girls.

This intervention provided us an exciting experience of working with students in the field of reproductive health. At the meetings that took place during the intervention with students, initially, we prioritized studying the childbearing and reproductive health knowledge among them, which is a matter of urgency. The lack of information that was not the purpose of this study was very much to be seen, so that many people in the control group, after completing the questionnaire in the first stage, without even intervention, had an increase in the score of the positive fertility motives, and perhaps the reason for this was the primary motivation for knowing through the questionnaire (Table II). Before this study, we had conducted a systematic review around the subject of intention of childbearing and some possible factors that could impact it. Whether or not these factors were effective, we included them in our questionnaire. Our justification was based on the fact that intention is a cultural and also individual issue (1-3) that can be changed by the influence of each or a group of these factors. The distribution of possible confounding factors was the same between the two groups at baseline analysis (Table I).

Because our main outcomes were evaluating the positive and negative motives “alteration through intervention,” let's go to the heart of matter. With a quick glance at Table II, we find that there are some areas of positive motivation (such as instrumental use of child, feeling the need for survival, and pleasure of childbearing) have lower scores than the other areas. More importantly, these scores at elementary level are lower than high school level. One of the most important reasons for this difference may be related to the difference in the basic knowledge of two groups of the participant on the fertility issues that have already been mentioned. So that, the lack of baseline analysis, student's knowledge was one of the big limitations of our study and the other Iranian studies (21, 22).

After the intervention was conducted in the best way, all positive scores were promoted whereas the negative ones were declined. On the other hand, No matter what really the participant's group was, their total intention score got better. “Satisfaction with parenting” was a domain that improved significantly more in both the children of elementary and high schools. Rubin and East introduced this domain as a well-known area that easily changed through education and could have worked to create positive fertility in young couples (23). It needs to be thought that this study, although not having a goal for parenting education, has been able to increase the score of individuals in this area. Therefore, it can be predicted that if interventions with accurate steps accomplished in this area can best increase the desired outcomes in terms of becoming ready for parenting and increasing the ability of parents to take care of their child. Another result of this study was that it showed that intervention increased the positive motivation and reduced the negative motivation in high school students more than the elementary ones. Hay ford and Agadjanian received to this fact that the education about this subject should be established in a proper level of knowledge. They suggested that “you can start from the elementary level but the suitable material for education must be available” (24). Among the negative motives, as mentioned earlier, our intervention in high school girls could properly decrease the score of negative motives. In this way, the most dominant score which was decreased belonged to child's care challenges. Khadivzadeh and colleagues mentioned this subject as the main barrier which stands in the way of childbearing among couples (22). Watanabe and Lee described “fears of responsibility for the children” as a factor that let adolescents escape from marriage (24). In this regard, Berrington and Pat taro believed that education about fertility desire must be focused on the fear, especially on the fear of being the parent and the responsibilities that come with it (23). One of the novelty of this study was the time of intervention. We did our performance before the participants experienced real marital relation and such pre-going interventions, if properly designed, could have the best effect on the formation of the right believes of the target groups.

We explored the behavior of childbearing motives in front of some factors that are mentioned in Table III. Totally, we found the great amount of relationship between the positive motives and religious beliefs in family. This relation was ascertained by some other authors (23, 24). Also, we explored a strong reverse correlation between mother's level of education and intention to have a child in their daughters. In the other side was a negative intention that had a strong straight correlation with mother's level of education and had similar but the reverse correlation with religious beliefs in family. These results were affirmed by some studies (22-24).

## 5. Conclusion

This study showed that we can improve our fundamental childbearing motives, even with small interventions. Our intervention could improve the positive childbearing motives among school girls. In this regard, the role of some confounding factors such as the religious beliefs in family and maternal education are the most important.

##  Conflict of Interest

The authors declare that they have no conflict of interest.
